# Personalized management of IBD; is there any practical approach?

**Published:** 2015

**Authors:** Mohsen Norouzinia, Nosratollah Naderi

**Affiliations:** *Gastroenterology and Liver Diseases Research Center, Shahid Beheshti University of Medical Sciences, Tehran, Iran*

Inflammatory bowel disease (IBD) is a chronic inflammatory disorder affecting the gastrointestinal tract and includes ulcerative colitis and Crohn’s disease. During last decades, the incidence of IBD has been increased, both in children and adults (1–3). While the precise etiology of IBD is unknown, IBD occurs due to an abnormal immune response to luminal antigens in genetically susceptible persons. Uhlig et al. classified the mechanisms of IBD into epithelial natural barrier and it’s response defects in bacterial phagocytosis, inflammatory process and immune system disorders, such as low antibody production, neutropenia or immune dysregulation (4). Although over 160 specific genetic loci have been identified underlying the predisposition, but Genome-wide association (GWA) studies are substantially improving our knowledge of the molecular pathways leading to IBD (5).

Genetic factors together can only explain a small proportion of hereditability and disease susceptibility (6). In monozygotic twins, the concordance rates for Crohn’s disease range between 20 and 50%, whereas the concordance rates for ulcerative colitis are even lower. IBD susceptibility is more prevalent in siblings of IBD patients than the general population; so occurence of IBD appears to be higher in family members(5,6).

Some environmental factors play major role in the pathogenesis of IBD. There are many epidemiologic changes between populations around the world but cannot be explained by genetic variations alone. The environmental triggers, such as dietary habits, improved socioeconomic status, improved sanitation, and different microbial exposures, all increase the risk of IBD.

Studies on immigrant populations can potentially help to dissect the etiologic significance of genetic and environmental factors in developing the disease. Moreover, first and second generation immigrants coming from low incidence to high incidence areas acquire levels of risk similar or higher than that of their adopted countries (7, 8). These findings suggest that the varying rates of IBD observed among racial and ethnic groups reproduce shared environmental influences (9). It appears that economical development of countries is associated with the increased risk of IBD (10, 11). It is clear that immigrants have a genetic back ground predisposition, there must be some environmental factors triggering disease expression. Environmental exposures during childhood periods are also important. On the other hand, IBD emerges when a society makes the transition economically from a ‘developing ‘to a ‘developed’ status so it is likely to be contributed by changes in the environment. Future studies in developing nations, such as Iran, may provide clues to our understanding of IBD. In addition to their effects on disease pathogenesis, genetic and environmental stimuli also influence composition of the intestinal microbiota (12, 13).

The molecular pathways of disease increase our knowledge in IBD to determine disease characteristics such as clinical appearance, therapeutic response and natural history. Ultimately, through the combination of genetic data and clinical with information about gene expression and environmental factors influences, like intestinal microbiota (14), it seems increasingly possible to reach at the peak in which assessments of disease course and treatment can be personalized for individual patients. Currently, many predicting factors have been suggested for IBD classification, prognosis and therapeutic strategies on the base of natural history, biologic and serologic markers, clinical evidence and genetics. The stability of genetic markers over time makes them more attractive candidates for use in predictive planning (15). Several studies have shown associations between immune response to intestinal microbial related antigens and recognition targets pattern (such as that encoded by NOD2) in patients with Crohn’s disease (16-20). Therefore, in spite of many recognized predisposing genetic loci, only limited number of genetic patterns such as NOD2, IL-12–IL-23 ,ATG16L1 , and also IL-10–IL-19 are shown to be effective in predicting diseases severity and classification of IBD (21). The practice of personalized management in IBD will probably use diagnostic algorithms, clinical characteristic, genetic, molecular and environmental markers in order to classify IBD patients in both therapeutic and prognosis directions.

## Conclusion

Similar to the other complex polygenic diseases, the paradigm of personalized IBD treatment will probably only be achieved if we try to integrate the genetic and environment factors, molecular mechanisms and immunological advances with insights to the gut microbiota pattern. Multiple stimulatory and regulatory processes are also involved in maintaining the balance between host defense and pathogenic inflammation gut epithelial. To date many pathways have been known clearly. Appropriate genetic diagnostic investigations can profoundly alter the chosen approaches, from immune suppression therapy and specific drugs for biological targets to allogeneic haematopoietic transplantation of stem cells. Future cohort studies across geographic social and ethnic groups will improve our data quality in suggesting multidimensional sequential panel for analyzing the patient information to approach practically personalized IBD care.
